# The role of fatty acid desaturase 2 in multiple tumor types revealed by bulk and single-cell transcriptomes

**DOI:** 10.1186/s12944-023-01789-0

**Published:** 2023-02-14

**Authors:** Enli Chen, Cong Wang, Hongwei Lv, Jing Yu

**Affiliations:** Cancer Center, Beijing Friendship Hospital, Capital Medical University, No. 95 Yong an Road, Beijing, 100053 Xi Cheng District China

**Keywords:** FADS2, Pan-cancer analysis, Biomarker, Prognosis, Immunotherapy

## Abstract

**Background:**

Previous studies have demonstrated the important role of fatty acid desaturase 2 (FADS2) in governing tumorigenesis and tumor metastasis. Although FADS2 is an essential regulator of fatty acid metabolism, its prognostic and immunotherapeutic value remains uncertain.

**Methods:**

The role of FADS2 was investigated across different types of tumors. Besides, the relationship between FADS2 and survival prognosis, clinicopathologic features, tumor-infiltrating immune cells, immunoregulatory genes, chemokines, chemokines receptor, tumor mutational burden (TMB), and microsatellite instability (MSI) was also explored. FADS2-related genes enrichment analysis was performed to further explore the molecular function of FADS2. Finally, the relationship between FADS2 expression and altered functional states in single-cell levels across different tumor cells was explored.

**Results:**

FADS2 was increased in most tumor tissues. Elevated FADS2 expression was associated with a poor overall survival (OS) and disease-free survival (DFS). FADS2 amplification was germane to worse progress-free survival (PFS). In addition, FADS2 correlated with the majority of tumor-infiltrating immune cells, immunoregulatory genes, and chemokines. Especially, FADS2 expression positively correlated with cancer-associated fibroblast (CAFs) infiltration. Gene Ontology and KEGG analysis demonstrated that FADS2 was involved in the fatty acid metabolic process, arachidonic acid metabolism, RAS, PPAR, and VEGF pathway. FADS2 had a positive relationship with tumor biological behaviors such as inflammation, cell cycle, proliferation, DNA damage, and DNA repair response in single-cell levels.

**Conclusions:**

FADS2 can serve as a potential prognostic and immunotherapeutic biomarker for multiple tumors, revealing new insights and evidence for cancer treatment.

**Supplementary Information:**

The online version contains supplementary material available at 10.1186/s12944-023-01789-0.

## Introduction

Tumor cells commonly undergo metabolic reconstruction, which supplies energy and building blocks for their growth, division, and survival [1–3]. Disruptions of glycolysis, amino acid, and lipid metabolism have been demonstrated to promote the growth of malignant tumors by causing cell proliferation, invasion, metastasis, and inhibiting apoptosis in previous studies [4–6]. Rewiring glycolipid metabolism is crucial in determining the mesenchymal phenotype of cancer cells and the faculty of those cells to resist death. Lipid breakdown provides tumor cells with enough building blocks and energy to synthesize cell membranes and perform other functions associated with proliferation. The plasticity of fatty acid metabolism has been largely recognized as a major factor affecting cancer progression and treatment efficacy [7, 8]. Based on these findings, new strategies are being explored by investigators to target lipid metabolism of cancer cells for the therapy of a broad variety of malignant tumors.

There is evidence that fatty acid desaturase 2 (FADS2) is a crucial enzyme in tumor cells involved in lipid metabolism [9–11]. Among the fatty acid desaturase (FADS) gene superfamily, stearoyl-CoA desaturase has been demonstrated to play a significant part in tumor malignant behaviors [12–14]. Dysregulation of the key enzyme FADS2 also directly interferes with fatty acid biosynthesis by tumor cells, which can hinder tumor progression. Arachidonic acid (AA) and its metabolites play a significant part in the cell's physiological process. Among them, the occurrence and progression of tumors can be reinforced by prostaglandin E2, leukotrienes, and cyclooxygenase 2 by diverse mechanisms. FADS2 is a key enzyme catalyzing such polyunsaturated fatty acids' production, and alterations in the expression and activity of FADS are linked to malignancy.

Though increasing numbers of studies has explored the potential role of FADS2 in the progression and treatment of cancer over the past decade, systematic pan-cancer studies are still lacking to fully demonstrate its oncogenic impact and prognostic value [15–18]. Thus, the objective of this study was to analyze the oncogenic impact and prognostic value of FADS2 across different types of tumors.

## Materials and methods

### Gene expression analysis of FADS2

The expression plot of FADS2 mRNA was conducted by the Human Protein Atlas (HPA). With the Tumor Immune Estimation Resource version 2.0 database (TIMER2.0) and The Cancer Genome Atlas (TCGA), FADS2 mRNA levels in tumor and normal tissues were compared across various tumors.

### Protein level analyses

The Clinical Proteomic Tumor Analysis Consortium (CPTAC) was used to determine the level of protein expression for FADS2 in numerous cancers. Based on the Human Protein Atlas, immunohistochemistry results of lung adenocarcinoma and renal cancer, as well as normal tissues were analyzed.

### Clinicopathologic features analyses

Gene Expression Profiling Interactive Analysis version 2 (GEPIA2.0) and CPTAC were utilized to analyze FADS2 mRNA and protein expression differences among different tumor stages. Besides, RNAseq and clinical tumor grade data were downloaded from the TCGA database to analyze whether the expression of FADS2 was related to the grade of tumor differentiation.

### Survival prognosis analyses

GEPIA 2.0 website was applied to construct overall survival (OS) and disease-free survival (DFS) plots of numerous tumors.

### Genetic alteration analyses

FADS2 genetic alterations were analyzed on the cBioPortal website. The frequency of FADS2 gene mutation, copy number alteration, and structural variant were carried out. Furthermore, the “Mutations” module was applied to construct a mutation site plot of FADS2. The survival plot was performed by separating cases according to the occurrence of FADS2 altered and unaltered groups.

### Analysis of immune cells infiltration

The “Immune” module of TIMER2.0, the Tumor Immune Dysfunction and Exclusion (TIDE), Extended Polydimensional Immunome Characterization (EPIC), MCPCOUNTER, and XCELL were used to explore the relationship between FADS2 expression and tumor-infiltrating immune cells. Furthermore, we combined FADS2 expression with the level of CAFs infiltration to assess the prognosis of patients.

### Correlation analyses of tumor mutational burden (TMB) and microsatellite instability (MSI) with FADS2

The genome assembly data was obtained from the UCSC website. MSI scores were computed for each tumor. Simple nucleotide variation datasets were downloaded and the TMB was calculated using R software package MAfTools. Finally, the TMB, MSI, and gene expression data of all these samples were integrated.

### FADS2-related genes enrichment

STRING was used to carry out a FADS2 co-expression network and we carried out Gene Ontology and KEGG pathway enrichment analysis of these 51 genes using the “clusterProfiler” R package (version: 3.13). The BioGRID4.3 database was also applied to carry out a FADS2-protein interaction network.

### Single cells sequencing

In CancerSEA, single-cell sequencing data can be analyzed at a single-cell level to obtain different functional status for cancer cells. We analyzed data from single-cell sequencing to determine whether FADS2 expression correlates with altered tumor functions. FADS2 expression profiles were shown in single cells across diverse tumor types using T-SNE diagrams.

## Results

### Gene expression analyses of FADS2

**Fig. 1:**
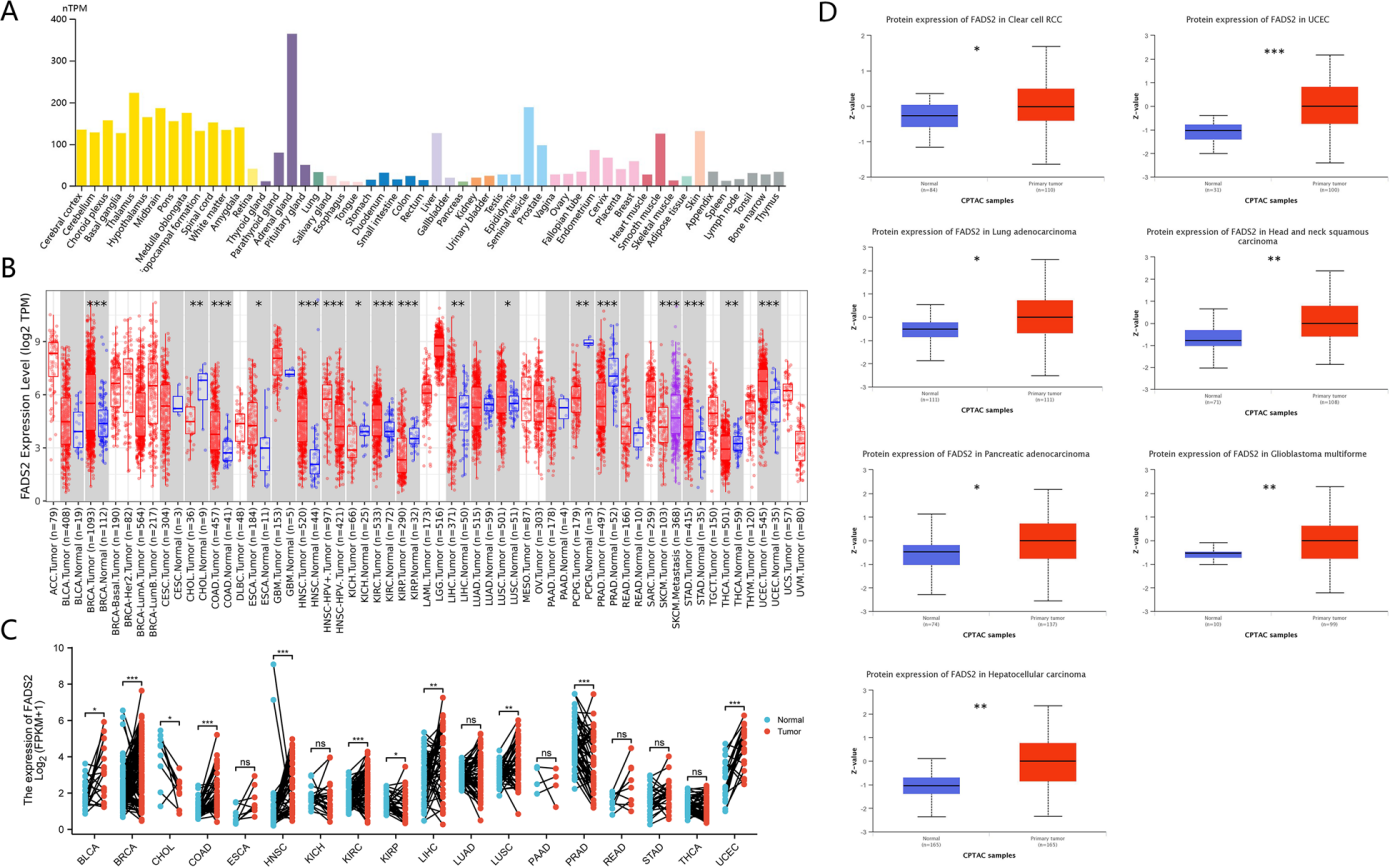
FADS2 expression status in different tumors and normal tissues. **A** Consensus FADS2 tissue expression based on datasets of HPA, GTEx, and FANTOM5. **B** The expression status of FADS2 in different tumor types was visualized by TIMER2. **C** Expression of FADS2 in different tumor types and paired adjacent normal tissues. **D** Expression of FADS2 protein in different tumor types and normal tissues. *p < 0.05; **p < 0.01; ***p < 0.001

FADS2 mRNA was highly expressed in the adrenal gland, thalamus, midbrain, medulla oblongata, liver, skin, seminal vesicle, and ductus deferens using the HPA, GTEx, and FANTOM5 datasets (see Fig. 1A and Supplementary Figure S1A-C). There was low tissue specificity for FADS2 mRNA expression based on these findings. FADS2 was relatively conservative in vertebrates (Supplementary Figure S1D). In head and neck squamous cell carcinoma (HNSC), esophageal carcinoma (ESCA), uterine corpus endometrial carcinoma (UCEC), colon adenocarcinoma (COAD), stomach adenocarcinoma (STAD), lung squamous cell carcinoma (LUSC), kidney renal clear cell carcinoma (KIRC), liver hepatocellular carcinoma (LIHC), and breast invasive carcinoma (BRCA), FADS2 mRNA level was elevated when compared to normal adjacent tissue (Fig. 1B). FADS2 mRNA expression was higher in HPV-HNSC than in HPV + HNSC (*P* < 0.05). Besides, the FADS2 mRNA expression level was also markedly higher in metastatic SKCM than in primary SKCM tissues (*P* < 0.05). However, FADS2 mRNA expression was markedly reduced in kidney chromophobe (KICH), cholangiocarcinoma (CHOL), prostate adenocarcinoma (PRAD), kidney renal papillary cell carcinoma (KIRP), and pheochromocytoma and paraganglioma (PCPG) (Fig. 1B). In addition, the TCGA database was used to validate the different FADS2 expression in tumors versus adjacent normal tissues. There was a significant increase in FADS2 mRNA expression in most types of cancer, such as UCEC, COAD, KIRC, BRCA, LUSC, HNSC, LIHC, and bladder urothelial carcinoma (BLCA) (Fig. 1C).

### Analyses of protein levels

**Fig. 2:**
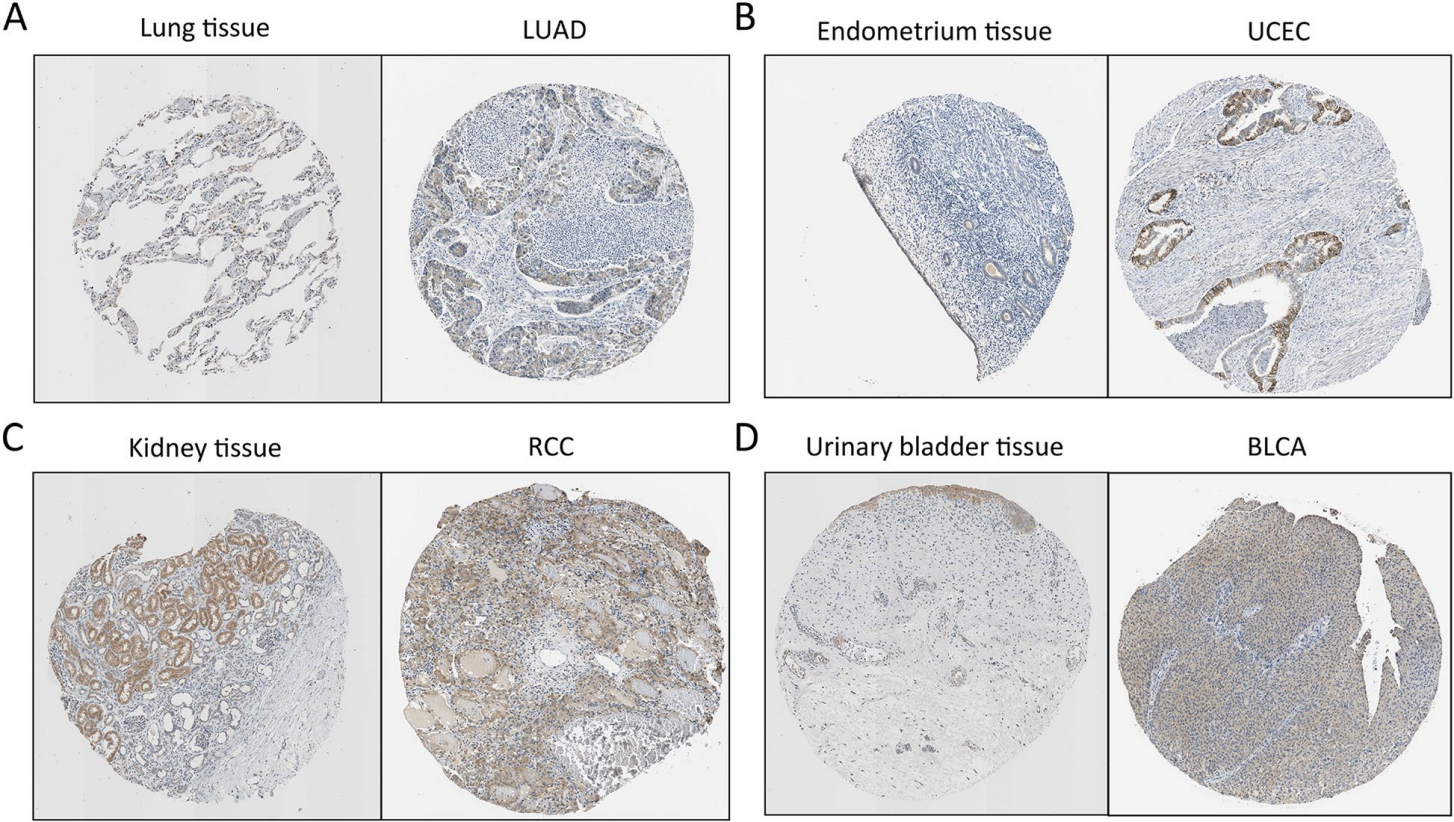
Representative images from HPA database illustrating immunohistochemistry staining of FADS2 for LUAD, UCEC, RCC, and BLCA were shown in Fig. 2A-D, respectively. Patients information: **A** Female, age 51, lung adenocarcinoma; **B** Female, age 79, endometrium adenocarcinoma; **C** Male, age 61, kidney adenocarcinoma; **D** Male, age 60, urothelial carcinoma, low grade

FADS2 protein levels increased in RCC, UCEC, LUAD, HNSC, PAAD, LIHC, and GBM (Fig. 1D). Representative images illustrating immunohistochemistry staining of FADS2 for LUAD, RCC, UCEC, and BLCA were shown in Fig. 2A-D.

### Clinicopathologic features of cancer patients and FADS2 expression

**Fig. 3:**
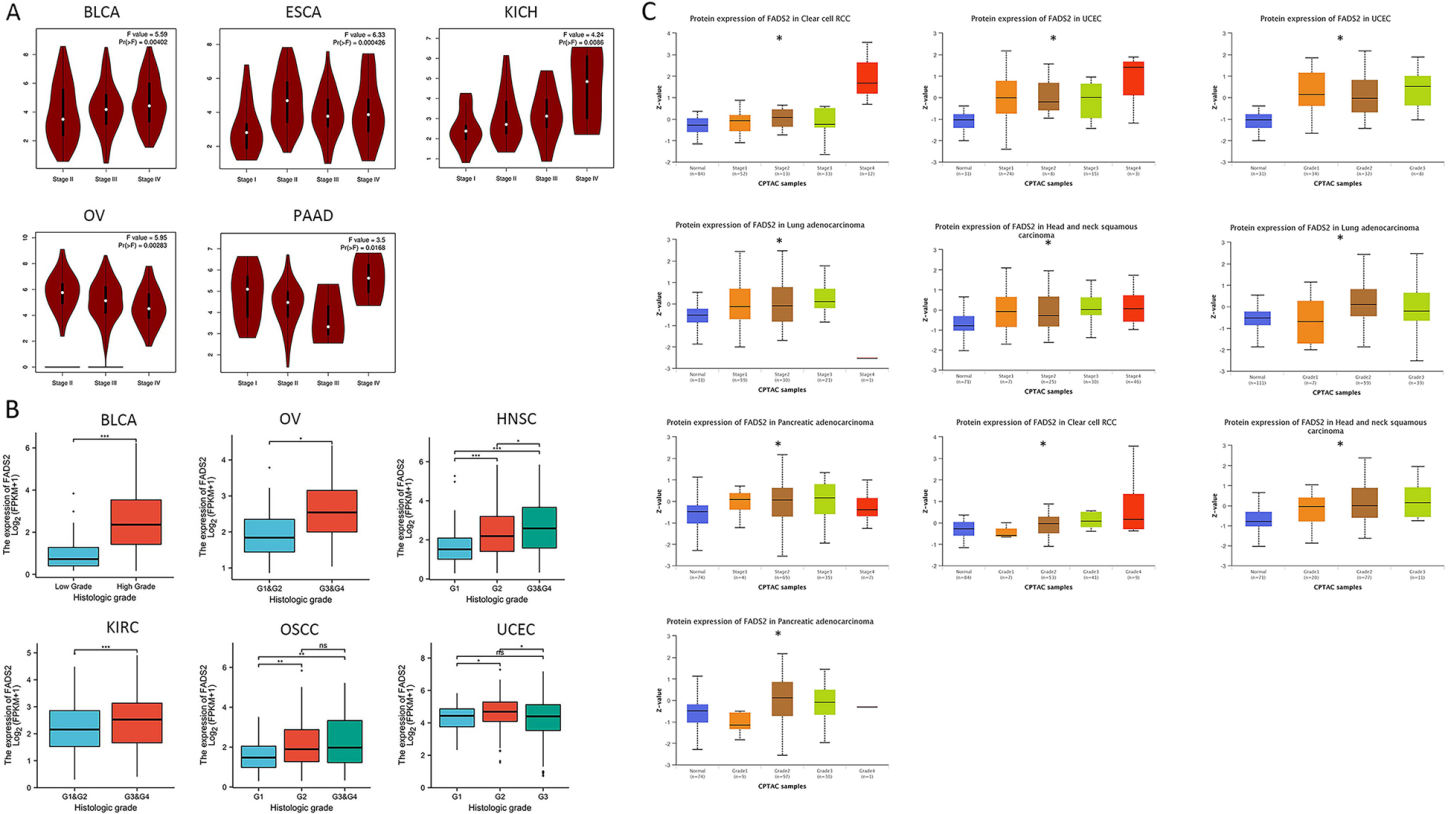
Correlation between FADS2 expression and pathological stages/histological grades of distinct tumors from TCGA and CPTAC datasets. **A** Correlation between FADS2 expression and pathological stages; **B** Correlation between FADS2 expression and histological grades; **C** Correlation between FADS2 protein expression and pathological stages/histological grades. *p < 0.05; **p < 0.01; ***p < 0.001

The relationship between FADS2 and tumor stages was investigated using GEPIA2. In tumor tissues of BLCA, ESCA, KICH, and PAAD, high FADS2 expression was rather associated with the advanced pathological stages (Fig. 3A). However, there was a negative correlation between FADS2 expression and pathological stage in OV. A positive correlation was found between FADS2 expression and histological grade in KIRC, OV, HNSC, and OSCC. At the protein level, FADS2 was positively linked to an advanced stage and higher histological grading in RCC, UCEC, LUAD, HNSC, and PAAD tumor tissues (Fig. 3B).

### FADS2 and patient prognosis

**Fig. 4:**
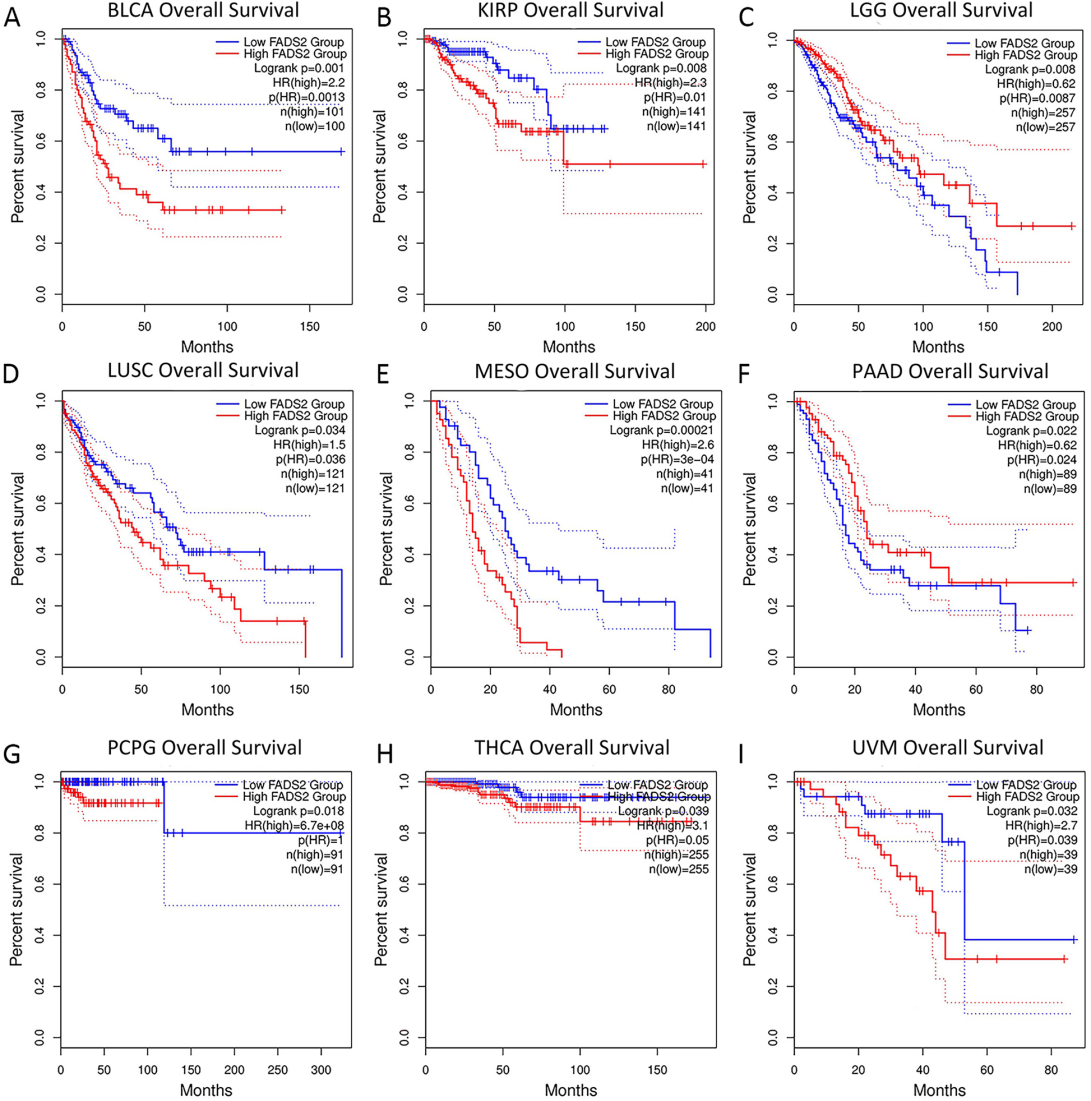
Correlation between FADS2 expression and overall survival in patients with various TCGA tumor types. **A**-**I** BLCA, KIRP, LGG, LUSC, MESO, PAAD, PCPG, THCA, and UVM, respectively. The Kaplan–Meier plots with significant results are displayed. The 95% confidence intervals of overall survival are indicated by red and blue dotted lines for high and low FADS2 groups, respectively

**Fig. 5:**
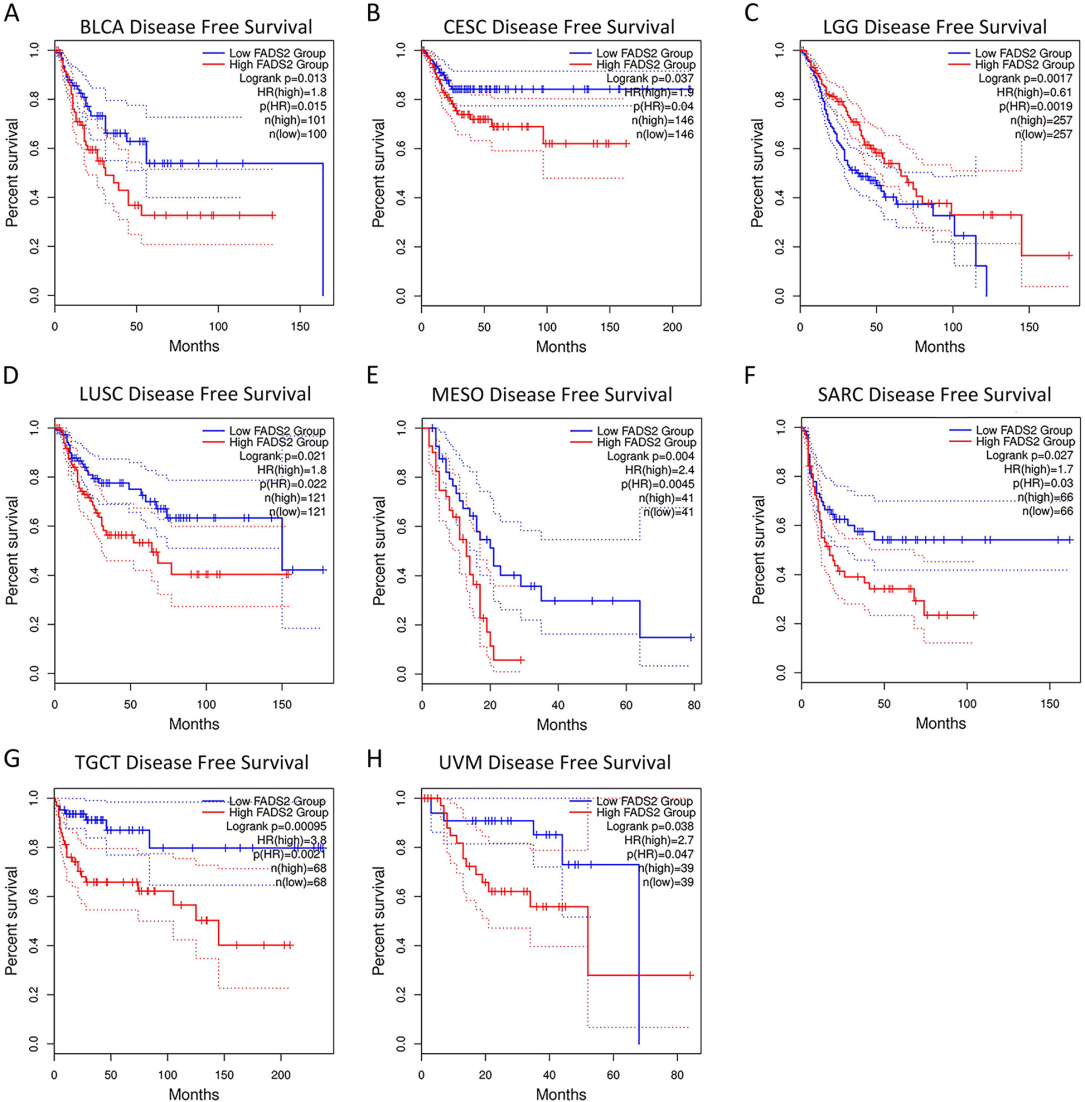
Correlation between FADS2 expression and disease-free survival in patients with various TCGA tumor types. **A**-**H** BLCA, CESC, LGG, LUSC, MESO, SARC, TGCT, and UVM, respectively. The Kaplan–Meier plots with significant results are displayed. The 95% confidence intervals of overall survival are indicated by red and blue dotted lines for high and low FADS2 groups, respectively

The relationship between FADS2 expression and prognosis such as OS and DFS of patients with various tumors was explored on the GEPIA2 website. A higher FADS2 expression has been associated with reduced OS in BLCA, KIRP, LUSC, PCPG, mesothelioma (MESO), thyroid carcinoma (THCA), and uveal melanoma (UVM) (Fig. 4). Moreover, the DFS plot indicated that higher FADS2 expression was associated with poorer prognosis in patients with BLCA, CESC, LUSC, MESO, SARC, TGCT, and UVM (Fig. 5). Nonetheless, higher FADS2 expression was related to improved OS in LGG and PAAD, and there was a significant correlation between FADS2 expression and a better DFS in LGG patients.

### Molecular changes in FADS2 in different tumor types

**Fig. 6:**
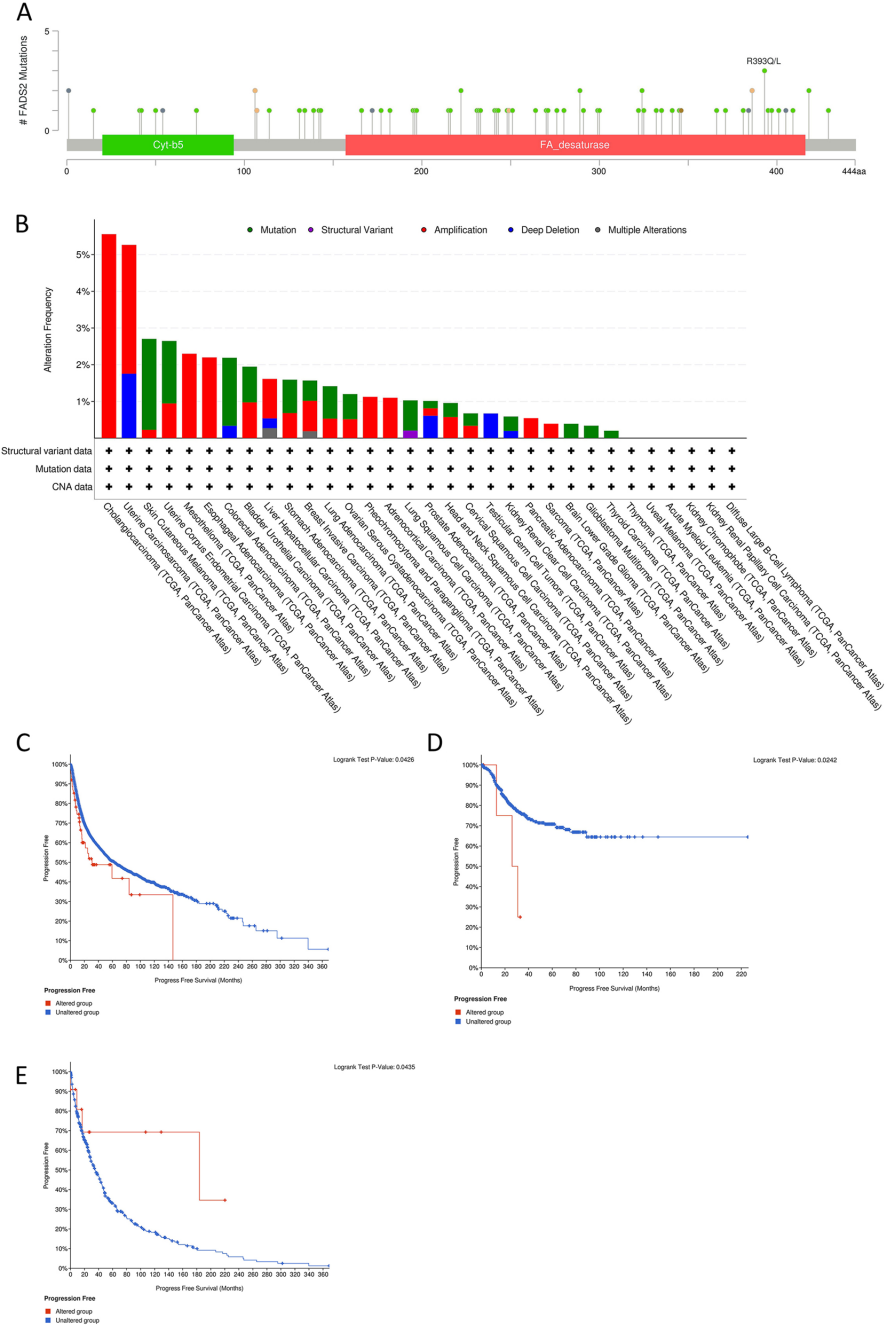
FADS2 genetic alteration in various tumor types of TCGA. **A** The alteration frequency with the mutation site of FADS2. **B** The alteration frequency with the FADS2 genetic alteration type were carried out by cBioPortal. **C** The correlations between FADS2 CNA status and progression-free survival of TCGA PanCancer Atlas (9 deep deletion; 55 amplification). **D** The correlations between FADS2 amplification and progression-free survival of patients with UCEC. (E)The correlations between FADS2 mutation and progression-free survival of patients with SKCM

In cBioPortal, the genetic modification of FADS2 in different tumor types was studied. In various tumor tissues, 77 FADS2 mutations were detected, including 60 missense mutations, 6 truncating mutations, 6 splice mutations, and 4 SV/fusion mutations and 1 in-frame mutation (Fig. 6A). FADS2 protein residue 393 was the most frequently mutated region with 3 mutations, making it the most commonly mutated region. Furthermore, there was the highest FADS2 genetic alteration frequency in CHOL and uterine carcinosarcoma tumor tissues (> 5%), and most of the genetic alterations were copy number amplification (Fig. 6B). Apart from CHOL and uterine carcinosarcoma tumor tissues, more than 2% of SKCM, UCEC, MESO, ESCA, and COAD tumor tissues showed genomic alteration of FADS2. Next, how FADS2 genetic alterations affect various types of tumors and their prognosis was discussed. FADS2 altered group (64 patients including 9 patients with deep deletion and 55 with copy number amplification) was associated with poorer PFS in all tumors (*P* = 0.043) (Fig. 6C). Especially, FADS2 amplification was relevant to worse PFS in patients with UCEC (*P* = 0.024) (Fig. 6D). Besides, SKCM patients with FADS2 mutations showed a better PFS (*P* = 0.044) (Fig. 6E).

### FADS2 correlates with the majority of tumor infiltrating immune cells, immunoregulatory genes and chemokines

**Fig. 7:**
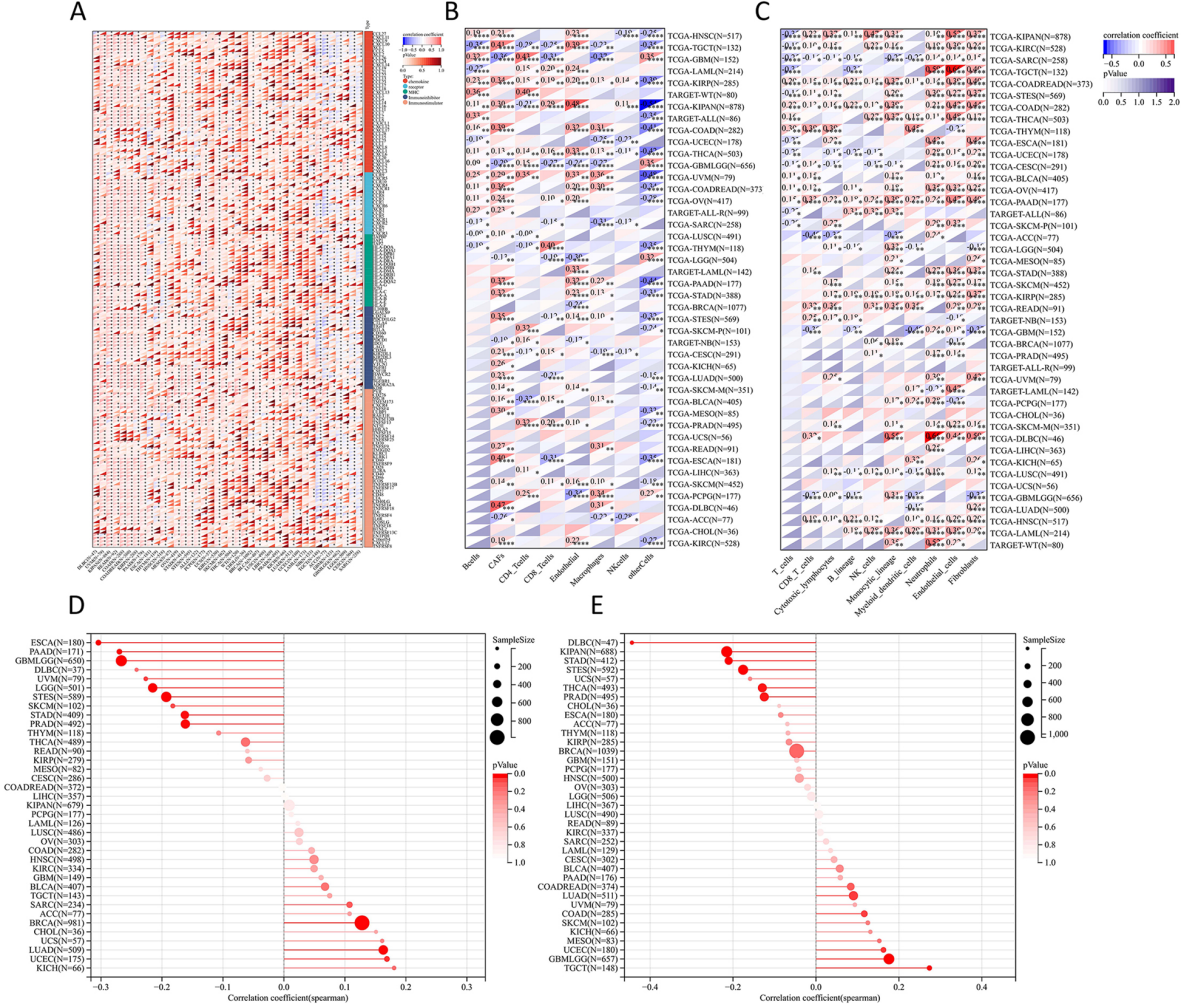
FADS2 correlate with immunoregulatory genes, chemokines, and chemokines receptor (**A**), tumor infiltrating immune cells (**B**-**C**), tumor mutational burden (TMB) (**D**), Microsatellite Instability (MSI) (**E**) in the majority of tumors

**Fig. 8:**
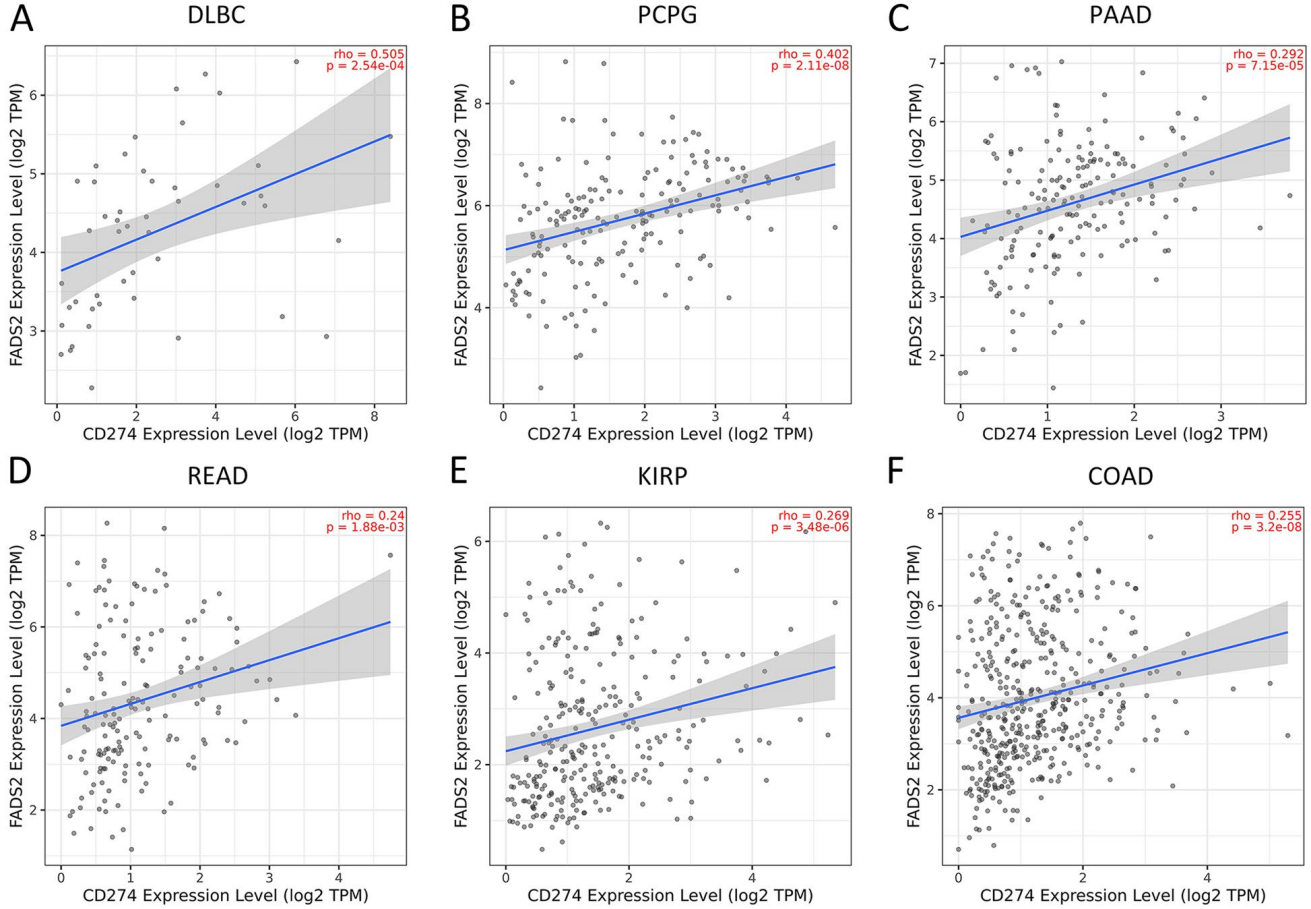
FADS2 positively correlated with PD-L1 (CD274) expression in DLBC (**A**), PCPG (**B**), PAAD (**C**), READ (**D**), KIRP (**E**), and COAD (**F**)

The tumor microenvironment is widely affected by immune-related cell infiltration, so the importance of FADS2 expression in pan-cancer immune infiltration analysis was further investigated. FADS2 was associated with infiltration levels of B cells in 19 cancer types, CD4 + T cells in 18 cancer types, CD8 + T cells in 18 cancer types, endothelial in 23 cancer types, macrophages cells in 20 cancer types, NK cells in 8 cancer types and CAFs in 30 cancer types according to the results of EPIC database (Fig. 7B). We confirmed the results in MCPcounter and XCELL, respectively (Fig. 7C and Supplementary Fig. 2). Three types of immune cells (neutrophils, endothelial cells, and CAFs) were positively correlated with FADS2 expression in most tumors. In summary, FADS2 may have a non-negligent role in tumor immunity. Thus, gene-level analysis of FADS2 was also carried out. Immune checkpoint genes, chemokines, chemokine receptors, and MHC in the majority of tumors were positively associated with FADS2, while negatively correlated in TGCT, ACC, GBM, GBMLGG, LGG, CESC, and SARC (Fig. 7A). Additionally, FADS2 positively correlated with PD-L1 (CD274) expression in DLBC, PCPG, PAAD, READ, KIRP, and COAD (Fig. 8). It could therefore be assumed that FADS2 was crucial for tumor immune regulation.

### CAFs infiltration analyses

**Fig. 9:**
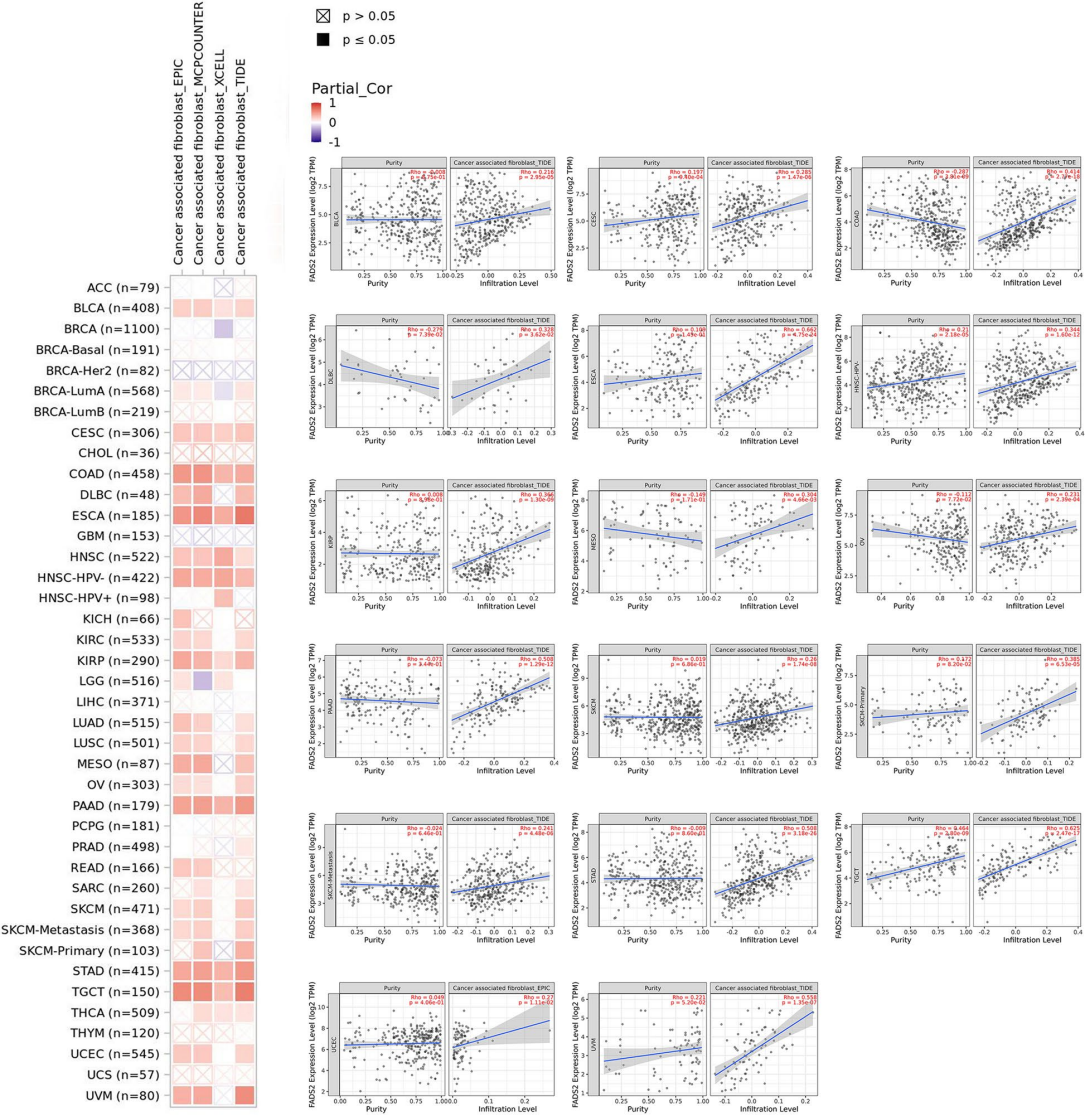
The expression of FADS2 was positively correlated with cancer-associated fibroblast infiltration in the majority of tumors

**Fig. 10:**
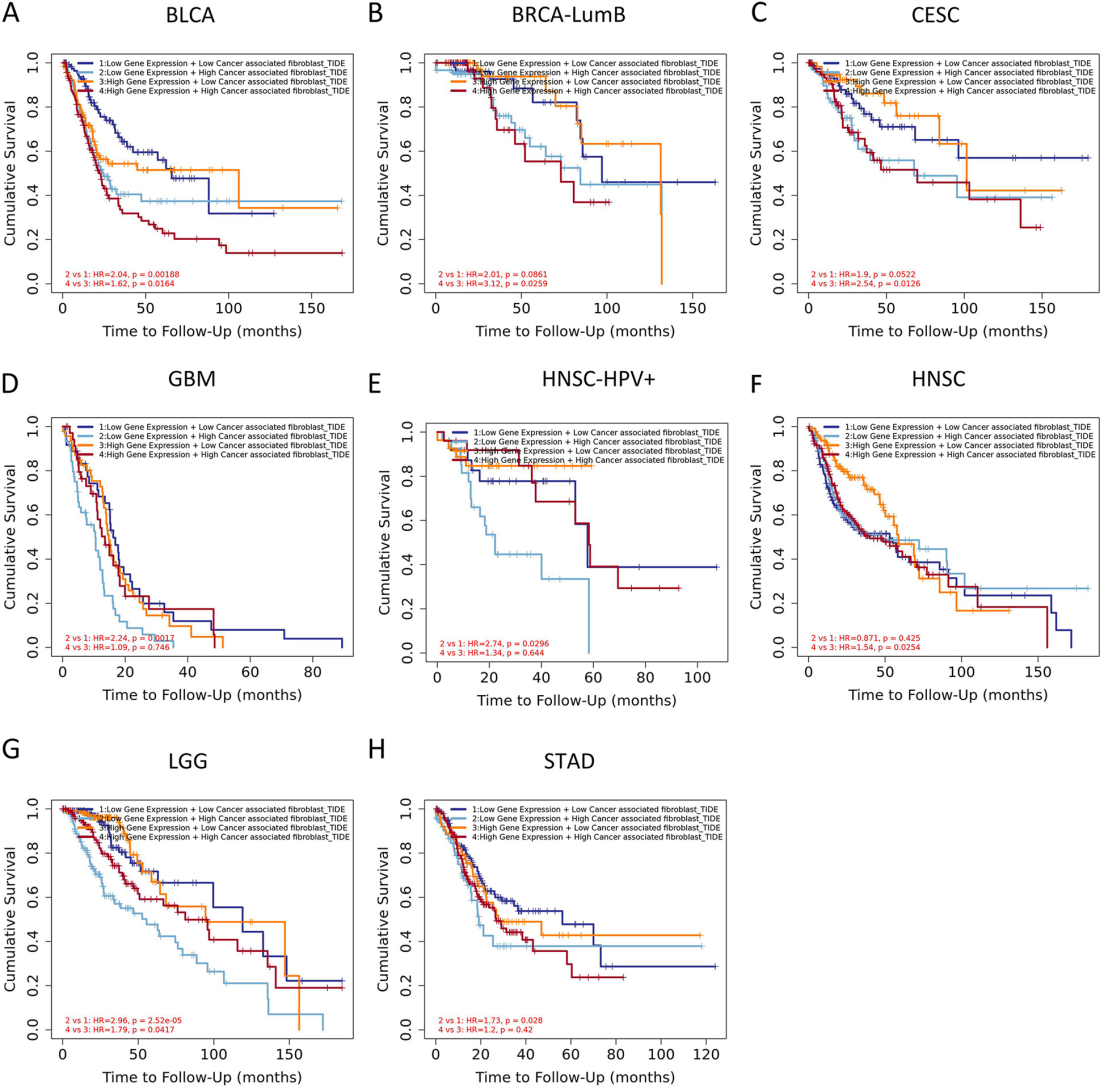
FADS2 expression combined with the level of cancer-associated fibroblast infiltration to assess the prognosis of patients. The Kaplan–Meier plots with significant results are displayed

EPIC, MCPcounter, XCELL, and TIDE were implemented to explore the association between FADS2 expression and CAFs infiltration in distinct tumor tissues. FADS2 was positively correlated with CAFs infiltration in SKCM, HNSC, BLCA, CESC, COAD, OV, DLBC, ESCA, KIRP, MESO, PAAD, STAD, TGCT, UCEC and UVM (Fig. 9). Moreover, we combined FADS2 expression with the level of CAFs infiltration to assess the prognosis of patients. High FADS2 expression and high CAFs infiltration were linked to a poorer prognosis of patients with BLCA, BRCA-LumB, CESC, and HNSC. However, for patients with GBM, HNSC-HPV + , and LGG, low FADS2 expression and high cancer-associated fibroblast cell infiltration indicated the worst prognosis (Fig. 10).

### FADS2 was associated with the TMB and MSI

The expression of FADS2 was positively related to TMB in UCEC, BRCA, and LUAD, whereas in GBMLGG、LGG、ESCA、STES、STAD、PRAD、PAAD, and UVM, it was adversely connected with TMB (Fig. 7D). In GBMLGG, LUAD, COAD, UCEC, and TGCT, FADS2 expression was inversely connected with MSI, but it was significantly corelated in STES, KIPAN, STAD, PRAD, THCA, and DLBC (Fig. 7E).

### FADS2-related GO and KEGG enrichment analysis

**Fig. 11:**
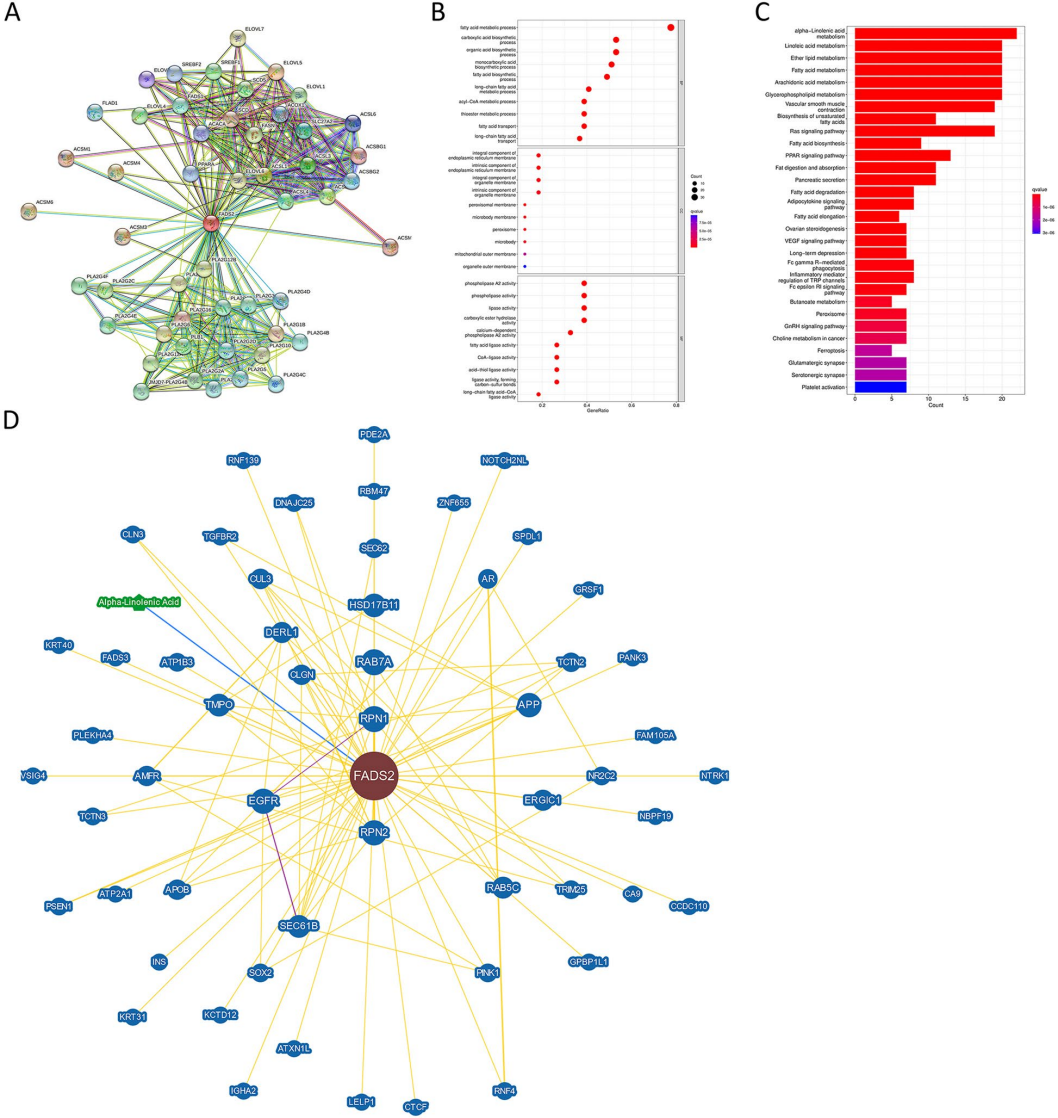
FADS2-related gene enrichment analysis. **A** PPI. **B **Gene Ontology (GO) analysis of genes obtained by the PPI. **C** KEGG analysis of of genes obtained by the PPI. **D** FADS2-Protein interactions obtained by BioGRID

**Fig. 12:**
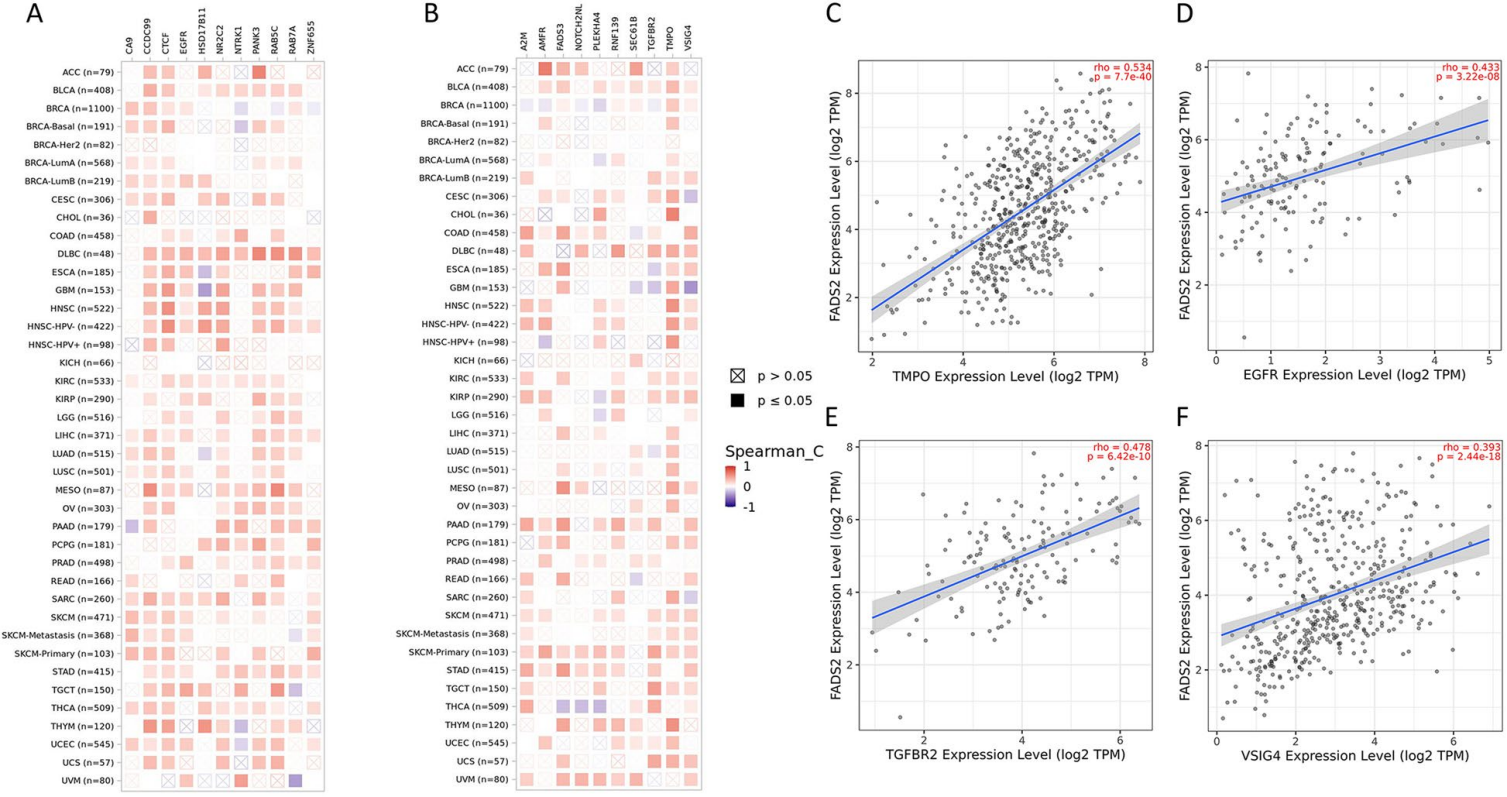
Correlation analysis between FADS2 and genes obtained by BioGRID conducted by TIMER 2.0 and GEPIA across all tumor samples from TCGA. **A**-**B** Heatmap of correlation analysis conducted by TIMER 2.0. **C** Correlation analysis between FADS2 and TMPO in HNSC. **D** Correlation analysis between FADS2 and EGFR in TGCT. **E** Correlation analysis between FADS2 and TGFBR2 in TGCT. **F** Correlation analysis between FADS2 and VSIG4 in COAD

To study the specific mechanisms of action of FADS2 in carcinogenesis, the STRING tool was used to extract the 50 genes actively interacting with FADS2 (Fig. 11A). Gene Ontology and KEGG enrichment analysis showed that these genes were closely associated with the fatty acid metabolic process, fatty acid biosynthetic process, monocarboxylic acid biosynthetic process, acyl-CoA metabolic process, integral component of endoplasmic reciculum membrane, peroxisomal membrane, α-linolenic acid metabolism, linoleic acid metabolism, arachidonic acid metabolism, vascular smooth muscle contraction, biosynthesis of unsaturated fatty acids, Ras signaling pathway, PPAR signaling pathway, VEGF signaling pathway, and ferroptosis (Fig. 11B-C). The findings motivated us to investigate whether FADS2 interacts with key proteins involved in the fatty acid metabolic process, VEGF, RAS, and PPAR signaling pathway to participate in these biological processes. The BioGRID4.3 database indicates that FADS2 interacts physically with RAB7A, RAB5C(RAS oncogene family members), NTRK1 (a member of the MAPK pathway), TGFBR2 (related to MAPK pathway), EGFR, SEC61B (involved in epidermal growth factor binding), CA9 (involved in cell proliferation and transformation), SPDL1(involved in cell cycle and cell migration), TMPO (involved in cell cycle control), VSIG4 (a strong negative regulator of T-cell proliferation and IL2 production), NR2C2 (involved in epithelial to mesenchymal transition) (Fig. 11). Aside from that, there was a correlation between the expression levels of FADS2 and the above genes, which further confirmed its vital role in the interaction of these genes (Fig. 12).

### Single-cell analyses of FADS2 expression

**Fig. 13:**
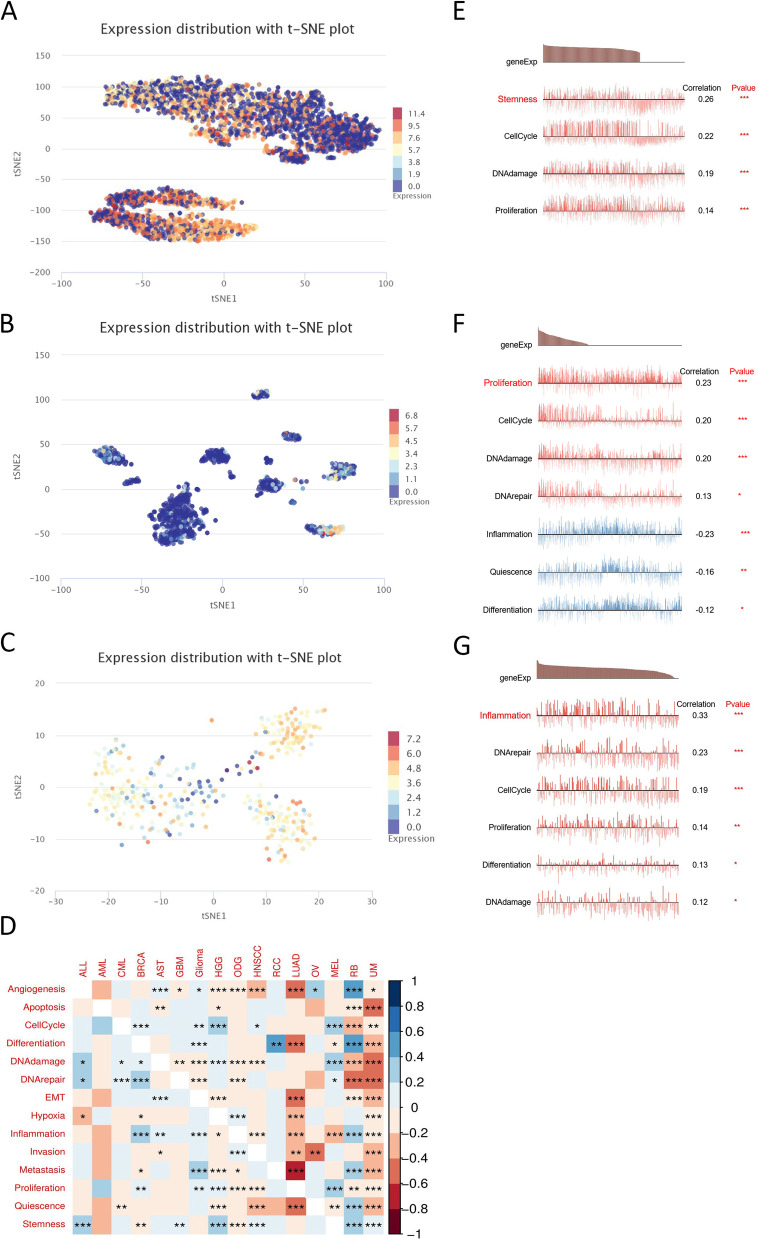
The expression levels of FADS2 at single-cell levels. FADS2 expression levels were shown at single cells from (**A**) Glioma, **B** Skin melanoma and (**C**) Breast cancer by T-SNE diagram. **D** The relationship between FADS2 expression and different functional states in single-cell levels across different tumor cells was explored by the CancerSEA tool. FADS2 expression and different functional states were shown at single cells from (**E**) Glioma, (**F**) Skin melanoma and (**G**) Breast cancer. *p < 0.05; **p < 0.01; ***p < 0.001

Sequencing the transcriptome of a single cell is a crucial technique used for analyzing the functions of candidate molecules in single cells. FADS2 expression profiles were demonstrated at single-cell levels from glioma, skin melanoma, and breast cancer by T-SNE diagram (Fig. 13A-C). In addition, the relationship between FADS2 expression and altered functional states in single-cell levels across different tumor cells was explored by the CancerSEA tool (Fig. 13D). In glioma, the expression of FADS2 was positively associated with stemness, cell cycle, DNA damage, and proliferation. FADS2 expression of breast cancer had a positive relationship with almost all tumor biological behaviors, such as inflammation, cell cycle, DNA repair response, proliferation, differentiation, and DNA damage. What's more, FADS2 expression was positively related to proliferation, cell cycle, DNA damage, and DNA repair response in skin melanoma. By contrast, FADS2 expression was negatively related to inflammation, quiescence, and differentiation in skin melanoma (Fig. 13E-G).

## Discussion

In this study, FADS2 was analyzed extensively in pan-cancer to determine its prognostic value and potential immunotherapy value. FADS2 expression was increased in both mRNA and protein and was associated with a worse OS and DFS in cancer patients. FADS2 was also correlated with TMB, MSI, and chemokines, as well as tumor-infiltrating immune cells. Especially, an association between FADS2 and PD-L1 expression was found to be significant. FADS2 expression was also noted to be positively linked to cancer-associated fibroblast (CAFs) infiltration in many cancers. The relationship between FADS2 expression and different functional states in single-cell levels showed that FADS2 expression had a positive relationship with tumor biological behaviors such as inflammation, cell cycle, proliferation, DNA damage, and DNA repair response in tumors. Accordingly, FADS2 could serve as a prognostic marker for pan-cancer and a biomarker for cancer immunologic infiltration.

The expression of both FADS2 mRNA and protein was elevated in the majority of tumors when compared to normal tissue. Furthermore, FADS2 mRNA expression was also significantly increased in most tumor tissues than in paired adjacent normal tissues. The above results were consistent with previous research results. In MDA-MB-231 cells compared to HMEC cells, Lin Q et al. observed an increased transcription of FADS2 [15]. In addition, Liu J et al. also indicated that FADS2 level was elevated in bladder cancer cell lines. In a microarray analysis of breast cancer and normal tissues, Zhao et al. discovered that the FADS2 mRNA level in cancer tissues was more than twice as high as in normal tissues [17]. He et al. indicated that the protein levels of FADS2, as well as its activity, in melanoma B16 tissues, were significantly higher than those of para-carcinoma tissues in a mouse model [18]. All of the results indicated that FADS2 were significantly up regulated in the majority of tumors. To sum up, it could be concluded that FADS2 may promote tumorigenesis and tumor metastasis in various tumors. Thus, it requires to further explore its clinical and prognosis significance.

Previous studies have reported the clinical and prognosis significance of FADS2 in tumors, but the type of tumors studied are only restricted to BLCA and LUSC [19–22]. Thus, we next investigated whether FADS2 over expression was associated with clinicopathological parameters and prognosis at the pan-cancer level. FADS2 over expression was associated with reduced OS and DFS in most types of tumors. However, in LGG, elevated FADS2 was associated with improved OS and DFS. The reason may be that LGG itself is sensitive to ferroptosis, while FADS2 can promote the synthesis of polyunsaturated fatty acids (PUFA) promoting the occurrence of ferroptosis, leading to a better prognosis in patients with high expression of FADS2 [23, 24]. High FADS2 expression was linked to the advanced pathological stage and histological grade across various cancers. FADS2 amplification was relevant to worse PFS in patients with UCEC. Consistently, SKCM patients with FADS2 mutations showed a better PFS. In summary, the association of FADS2 with increased risk of progression and death in the majority of tumors suggested a tumor-promoting role.

Numerous prior research has been carried out on the molecular function of FADS2 for cancers. Xuan Y et al. performed lipidomic analysis and showed that the increase of unsaturated fatty acids was positively linked to FADS2 levels and the oncogenic capacities of ovarian cancer cells. However, inhibition and genetic ablation of FADS2 suppress tumor growth [25]. Using FADS2 knockdown, Jiang et al. showed that lung cancer growth was significantly suppressed, thereby increasing Fe and lipid reactive oxygen species levels and significantly reduced in the levels of ferroptosis-related genes, which ultimately lead to ferroptosis [26]. Wang et al. treated U-87MG and LN-229 cells under radiotherapy in vitro with SC-26196 (FADS2 inhibitor) and showed a 45% proliferation rate reduction, a 41% colony formation reduction, and a 30%-40% apoptosis increase. By blocking the synthesis of AA and PGE2, SC26196 reverses radioresistance in PGE2-ID1-dependent glioblastomas [27]. Despite these findings, research on the molecular function of FADS2 is still lacking. Heretofore, the precise mechanism of how FADS2 exerts its tumor-promoting function and the effects on the tumor immunological microenvironment is still unclear.

There is an extensive interrelationship between immune cells and cancer cells and they play a vital role in cancer migration and metastasis. It has been indicated that CAFs can promote tumor growth, angiogenesis, and metastases and be linked to a poor prognosis, chemotherapy resistance, and recurrence of cancer [28, 29]. FADS2 expression correlates positively with CAFs infiltration. We speculated that the presence of FADS2 could facilitate the formation and infiltration of CAFs. In addition, high FADS2 expression and high CAF cell infiltration was related to a poorer prognosis of patients with BLCA, BRCA-LumB, CESC, and HNSC. In summary, this study revealed FADS2's role in the tumor immune microenvironment and its prognostic value for numerous cancers.

A growing body of research has shown that there is a correlation between tumor lipid metabolism and tumor immunotherapy. A synergistic immune response can be induced by targeting lipid metabolism and immune checkpoint inhibitors [30, 31]. FADS2 is an important component of lipid metabolism, and it is a downstream target of the SCAP/SREBP pathway that regulates lipid synthesis in cells. Lim SA et al. reported that the synthesis of lipids promoted maturation of Tregs and improved PD-1 expression to suppress tumor immunity, which could be further upregulated through the SCAP/SREBPs pathway [32]. The results showed a statistical correlation between FADS2 and PD-L1 expression in various tumors.

Consistently, Yang Y et al. have reported that the synthesis of lipids could increase PD-L1 expression and promote tumor immunosuppression through palmitoylation of PD-L1 in BRCA [33]. Combined with these findings, we speculate that FADS2 plays a tumor immune-suppressive role might be partly by promoting the expression of PD-L1 in tumor cells. Further, FADS2 expression was positively correlated with TMB and MSI in most tumors. The above studies indicated that FADS2 might be a promising immunotherapy biomarker.

Through the results of Gene Ontology and KEGG enrichment analysis, FADS2 plays an oncogenic driver role by driving cancer cells proliferation, cell migration, and EMT through VEGF, MAPK, and PPAR pathways. An analysis of GSEA by Zhu K et al. revealed that the PPAR signature correlated with the infiltration of tumor immune cells [21]. In a recent study, Mokhtari et al. showed a cross-talk between the unsaturated fatty acid synthesis pathway and arachidonic acid metabolism and that the PPAR signaling pathway was regulated via these pathways. Besides, migration, colony formation, and proliferation of HT-29 cells decreased after adding linoleic acid [34]. The findings were in agreement with these studies. Besides, FADS2 might play an immunosuppressive effect by interacting with VSIG4 to suppress T-cell proliferation and IL2 production, and by interacting with TGF-βR2, CA9, NR2C2 to stimulate fibroblast, bone marrow mesenchymal stem cells, and epithelial cells to convert into CAFs through TGF-β and EMT. Finally, the relationship between FADS2 expression and altered functional states in single-cell levels across different tumor cells was explored by the CancerSEA. FADS2 expression had a positive relationship with tumor biological behaviors such as inflammation, cell cycle, proliferation, DNA damage, and DNA repair response in tumors. In summary, FADS2 can serve as a potential prognostic and immunotherapeutic biomarker for multiple tumors.

### Comparisons with other studies and what does the current work add to the existing knowledge

The study demonstrated for the first time that FADS2 was associated with PD-L1 expression, TMB, and MSI. Besides, FADS2 correlated with the majority of tumor-infiltrating immune cells (especially CAFs), immunoregulatory genes, and chemokines, revealing potential cancer-promoting mechanisms of FADS2.

### Study strength and limitations

In addition, FADS2 expression positively correlated with CAFs infiltration; FADS2 was associated with PD-L1 expression, TMB, and MSI. In single-cell levels, FADS2 expression had a positive relationship with tumor biological behaviors such as inflammation, cell cycle, proliferation, DNA damage, and DNA repair response in tumors. These results suggested that FADS2 could serve as a potential prognostic and immunotherapeutic biomarker for multiple tumors. The current research had some limitations. We concluded only through data mining from publicly available databases. This study lacked experimental verification, and we will carry out future experiments. To compensate for this and to make the conclusion more credible now, we performed a comprehensive analysis of proteomics and transcriptomics (bulk and single-cell sequencing) to validate the role of FADS2 in cancers.

## Conclusions

As a result of these findings, it is concluded that FADS2 is frequently overexpressed in the majority of tumors and that its expression level and genetic alteration are significantly related to the prognosis of cancer patients. Additionally, FADS2-related gene enrichment analysis and immune infiltration analysis provide evidence of FADS2's potential role in regulating tumor immunity and indicate FADS2 can serve as a potential prognostic and immunotherapeutic biomarker for patients with cancers. Therefore, FADS2 may become a potential therapeutic target for cancer treatment.

## Supplementary Information

**Additional file 1: Figure S1.** FADS2 mRNA was highly expressed in the adrenal gland, thalamus, midbrain, medulla oblongata, liver, skin, seminal vesicle and ductus deferens using the HPA (A), GTEx (B), and FANTOM5 (C) datasets. FADS2 was relatively conservative in vertebrates (D).

**Additional file 2: Figure S2.** FADS2 correlate with the majority of tumor infiltrating immune cells utilizing XCELL.

## Data Availability

The datasets presented in this study can be found in online repositories. The names of the repository/repositories can be found in the article.

## Abbreviations

FADS2: Fatty acid desaturase 2
TMB: Tumor mutational burden
MSI: Microsatellite instability
OS: Overall survival time
DFS: Disease-free survival
PFS: Progress-free survival
AA: Arachidonic acid
SCD: Stearoyl-CoA desaturase
EMT: Epithelial–mesenchymal transition
HPA: The Human Protein Atlas database
TIMER2.0: The Tumor Immune Estimation Resource version 2.0 database
TCGA: The Cancer Genome Atlas database
CPTAC: The Clinical Proteomic Tumor Analysis Consortium
GEPIA2.0: Gene Expression Profiling Interactive Analysis version 2
TIDE: The Tumor Immune Dysfunction and Exclusion
EPIC: Extended Polydimensional Immunome Characterization
LAML: Acute Myeloid Leukemia
BLCA: Bladder Urothelial Carcinoma
BRCA: Breast invasive carcinoma
CESC: Cervical squamous cell carcinoma and endocervical adenocarcinoma
CHOL: Cholangiocarcinoma
COAD: Colon adenocarcinoma
ESCA: Esophageal carcinoma
GBM: Glioblastoma multiforme
HNSC: Head and Neck squamous cell carcinoma
KICH: Kidney Chromophobe
KIRC: Kidney renal clear cell carcinoma
KIRP: Kidney renal papillary cell carcinoma
LIHC: Liver hepatocellular carcinoma
LGG: Lower Grade Glioma
LUAD: Lung adenocarcinoma
LUSC: Lung squamous cell carcinoma
MESO: Mesothelioma
OV: Ovarian serous cystadenocarcinoma
PAAD: Pancreatic adenocarcinoma
PCPG: Pheochromocytoma and Paraganglioma
PRAD: Prostate adenocarcinoma
READ: Rectum adenocarcinoma
SARC: Sarcoma
SKCM: Skin Cutaneous Melanoma
STAD: Stomach adenocarcinoma
TGCT: Testicular Germ Cell Tumor
THYM: Thymoma
THCA: Thyroid carcinoma
UCS: Uterine Carcinosarcoma
UCEC: Uterine Corpus Endometrial Carcinoma
UVM: Uveal Melanoma
