# Supplementary figures and images for: The role of fatty acid desaturase 2 in multiple tumor types revealed by bulk and single-cell transcriptomes
Source: Lipids Health Dis. 2023 Feb 14;22:25. doi: 10.1186/s12944-023-01789-0 (PMC9930218; doi:10.1186/s12944-023-01789-0)

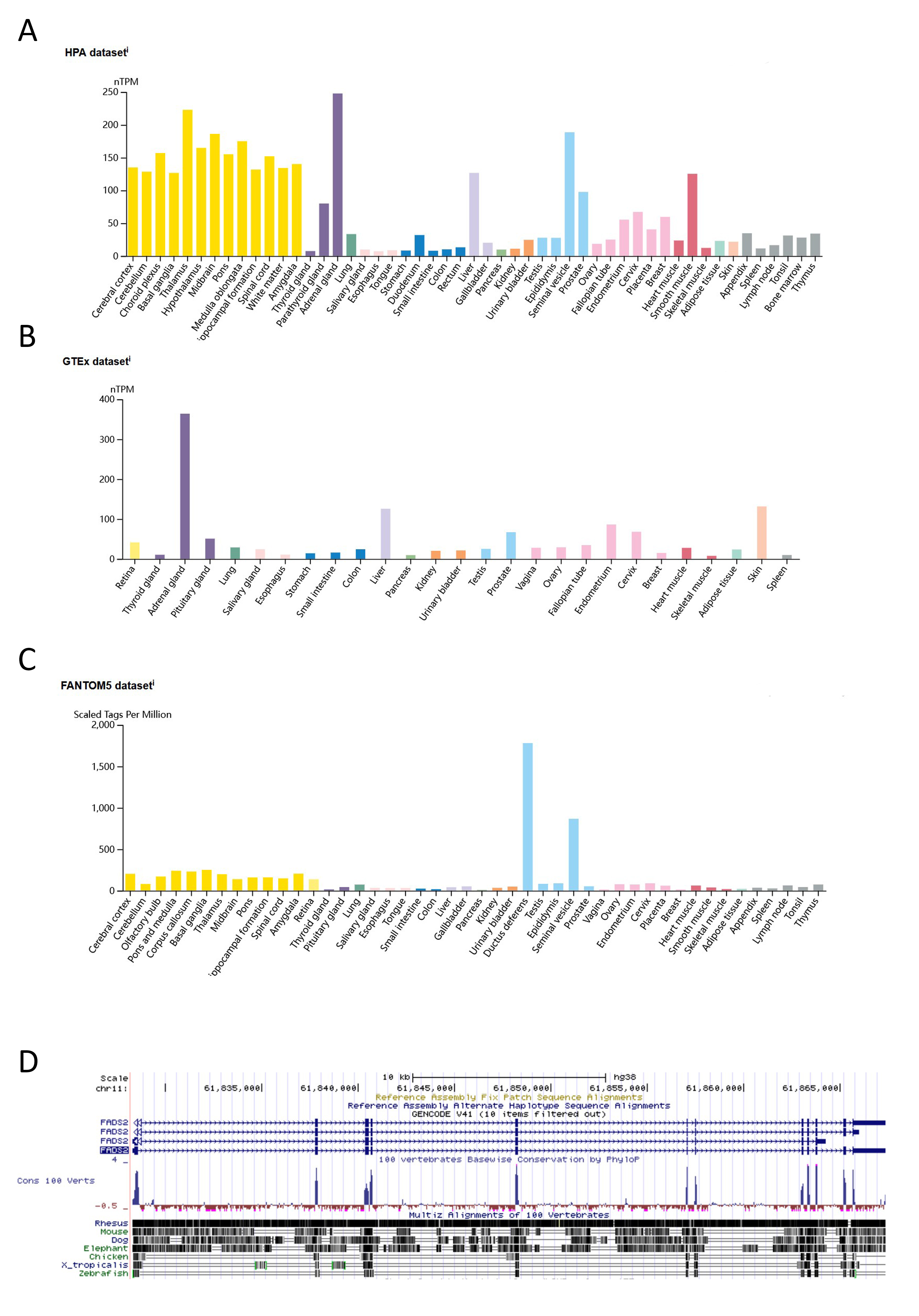

Supplement: Supplementary file 1 — Additional file 1: Figure S1. FADS2 mRNA was highly expressed in the adrenal gland, thalamus, midbrain, medulla oblongata, liver, skin, seminal vesicle and ductus deferens using the HPA (A), GTEx (B), and FANTOM5 (C) datasets. FADS2 was relatively conservative in vertebrates (D). [file 12944_2023_1789_MOESM1_ESM.jpg]

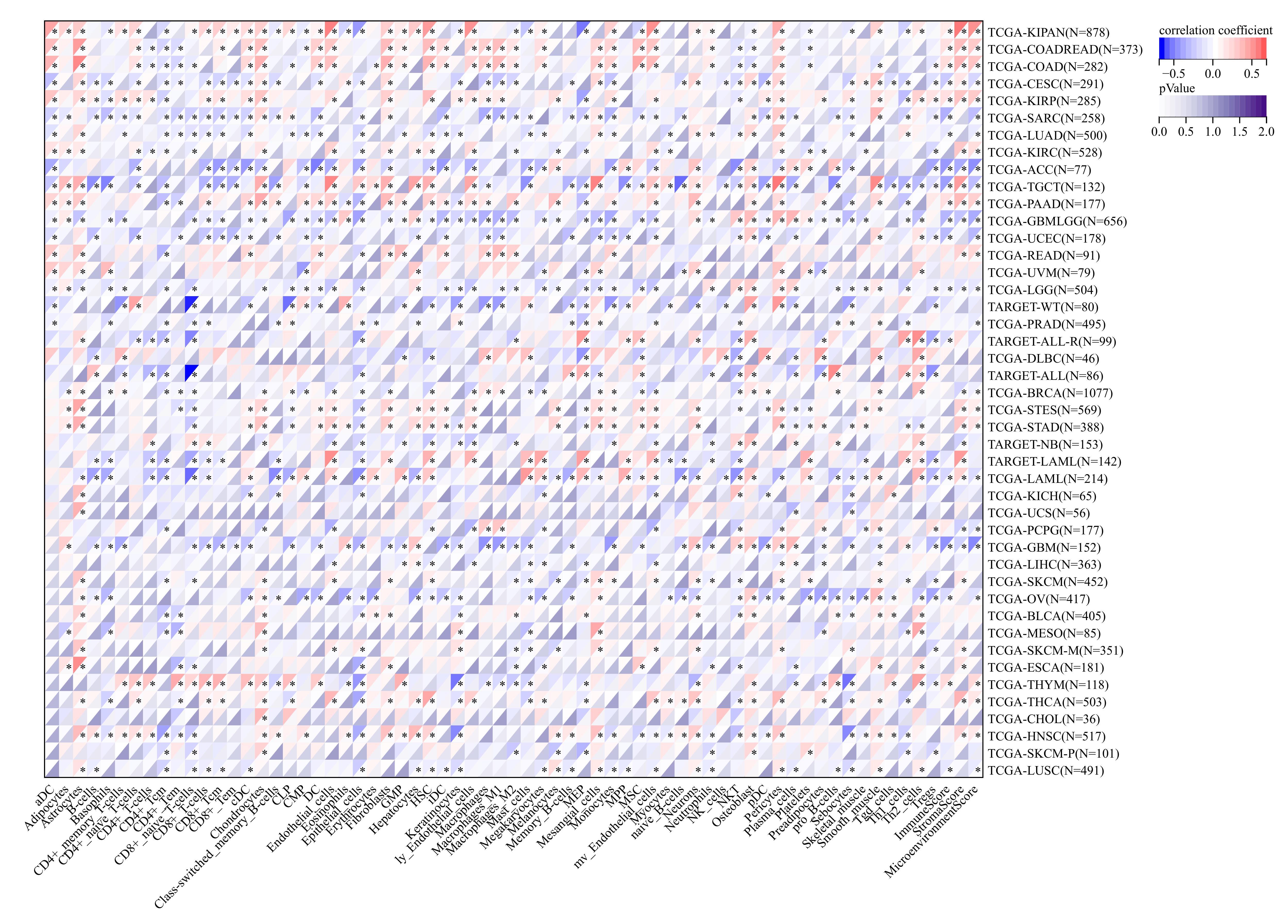

Supplement: Supplementary file 2 — Additional file 2: Figure S2. FADS2 correlate with the majority of tumor infiltrating immune cells utilizing XCELL. [file 12944_2023_1789_MOESM2_ESM.jpg]
